# Association between Blood Pressure and Post-Stroke Cognitive Impairment: A Meta-Analysis

**DOI:** 10.31083/j.rcm2505174

**Published:** 2024-05-16

**Authors:** Huifen Huang, Yanli Zhan, Linling Yu, Shan Li, Xueli Cai

**Affiliations:** ^1^Neurology Department of Lishui Municipal Central Hospital, 323000 Lishui, Zhejiang, China; ^2^Lishui Cardio-Cerebrovascular Disease Prevention Center, 323000 Lishui, Zhejiang, China

**Keywords:** post-stroke cognitive impairment, vascular cognitive impairment, stroke, blood pressure, hypertension, meta-analysis

## Abstract

**Background:**

Post-stroke cognitive impairment (PSCI) represents a serious post-stroke complication with poor cognitive consequences. A vascular consequence after a stroke is that the occurrence and progression of PSCI may be closely related to blood pressure (BP). Thus, we systematically reviewed and performed a meta-analysis of the literature to examine the correlations between BP and PSCI.

**Methods:**

We systematically queried databases, including PubMed, the Cochrane Library, Embase, and Scopus, and conducted meta-analyses on studies reporting odds ratios (ORs) related to the association between BP and PSCI. Two authors autonomously assessed all titles, abstracts, and full texts and extracted data following the Meta-Analysis of Observational Studies in Epidemiology guidelines. The quality of the studies was evaluated using the modified Newcastle–Ottawa scale.

**Results:**

Meta-analyses incorporated 12 articles comprising a cumulative participant cohort of 21,732 individuals. The quality assessment indicated good in five studies, fair in one study, and poor in six. Through meta-analyses, we found that hypertension, systolic or diastolic BP (SBP or DBP) was significantly associated with PSCI (OR 1.53, 95% confidence interval (CI), 1.18–1.99; *p* = 0.001, *$I^{2}$* = 66%; OR 1.13, 95% CI, 1.05–1.23; *p* = 0.002, *$I^{2}$* = 52%; OR 1.38, 95% CI, 1.11–1.72; *p* = 0.004, *$I^{2}$* = 90%, respectively). In the subgroup analysis, SBP <120 mmHg, 120–139 mmHg, 140–159 mmHg, 160–179 mmHg, and DBP ≥100 mmHg highly predicted the occurrence of PSCI (OR 1.15, *p* = 0.0003; OR 1.26, *p* = 0.010; OR 1.15, *p* = 0.05; OR 1.02, *p* = 0.009; OR 1.96, *p* < 0.00001, respectively). However, the predictive effect of BP for PSCI declines when SBP ≥180 mmHg and DBP ≤99 mmHg (*p* > 0.05). Statistical heterogeneity was moderate to high, and publication bias was detected in SBP for PSCI.

**Conclusions:**

Considering the multifactorial etiology of PSCI, it is difficult to conclude that BP is an independent risk factor for PSCI. Given the restricted inclusion of studies, caution is advised when interpreting the findings from this meta-analysis. Subsequent investigations with substantial sample sizes are essential to exploring BP as a prospective target for addressing PSCI.

**Trial Registration Number:**

CRD42023437783 from PROSPERO.

## 1. Introduction

Post-stroke cognitive impairment (PSCI) is a cognitive dysfunction or decline that occurs following a stroke and manifests as deterioration in orientation, memory, and executive function [1]. Moreover, the onset of PSCI has no specific time frame. The present study suggests that PSCI may manifest between 3 and 6 months post-incident stroke, exhibiting a potential stabilization within the initial 12 months. However, it is also accepted that PSCI may persist or advance in the years following a stroke [1, 2]. In some stroke survivors, PSCI could cause severe complications or comorbidities, such as incontinence, depression, and dementia. These complications tend to bring extreme distress to patients and caregivers. Cognitive impairment is observed in 20%–80% of post-stroke individuals, displaying variability based on geographic location, racial demographics, and diagnostic criteria [3]. In addition, some risk factors also predispose stroke patients to PSCI, including older age, diabetes, atrial fibrillation, small vascular diseases (SVDs), etc. [4, 5, 6]. Among these risk factors, blood pressure (BP) may also contribute to the occurrence and progression of PSCI [7].

The mechanism of BP on PSCI is complex and unclear; the consideration of treating vascular risk has been proposed as a potential preventive measure for cognitive impairment [8]. Furthermore, previous studies also found that abnormal BP at baseline or follow-ups was a risk factor for PSCI [3, 9]. In other words, hyper- and hypo-BP may increase the risk of cognitive impairment in patients following a stroke, which is a U-shaped relationship that has been reported in other articles [10, 11, 12]. Indeed, several studies have detailed a positive association between BP and PSCI [13, 14], although these results are inconsistent [5, 8, 15]. Therefore, we performed a meta-analysis to quantify the association between BP and PSCI and to provide a definite threshold if possible.

## 2. Methods

The protocol of this systematic review was registered with the International Prospective Register of Systematic Reviews (registration no. CRD 42023437783), following the Meta-Analysis of Observational Studies in Epidemiology (MOOSE) guidelines [16].

### 2.1 Data Sources and Search Strategy

Two independent reviewers systematically searched PubMed, the Cochrane Library, Embase, and Scopus, with no restrictions on publication date. For publication language, only articles published in English were considered and included. Moreover, a manual search of reference lists for related reviews and studies was conducted. The search string was built as follows: (“blood pressure” OR hypertension OR hypotension) AND (“cognitive impairment” OR “cognitive decline” OR dementia) AND (stroke). A **Supplementary File** (Supplementary: Search key terms and strategy) describing the comprehensive search term framework is attached.

### 2.2 Eligibility Criteria

The systematic review adhered to the PECOS (P-participants, E-exposure, C-comparisons, O-outcomes, S-study design) framework for identifying potential eligible studies. The inclusion and eligibility criteria were defined as follows:

(1) Type of participants: Studies were included if participants were adults (≥18 years) with cognitive impairment after stroke, including ischemic or hemorrhagic stroke. Participants with other cognitive dysfunction diseases (e.g., Alzheimer’s disease, Parkinson’s disease, psychiatric disorders) were excluded.

(2) Type of exposures: Studies examined systolic or diastolic BP (SBP or DBP), or both, hypertension, or hypotension were included. Studies that focused on BP variability or pulse pressure were excluded.

(3) Type of comparisons: There was no restriction on comparisons.

(4) Type of outcomes: Cognitive impairment should be operationalized or assessed using standardized instruments, including but not limited to the Mini-Mental State Examination (MMSE), Montreal Cognitive Assessment (MoCA), Cambridge Cognition Examination (CAMCOG), etc. [1].

(5) Type of study designs: Observational studies (e.g., prospective or retrospective, and cross-sectional studies) or post-hoc or secondary analyses from randomized controlled trial (RCT) reporting the odds ratio (OR) of PSCI were included.

### 2.3 Study Selection

All retrieved records were initially exported to EndNote X9 (version EndNote X9.3.2, Captivate Analytics, San Francisco, CA, USA) for deduplication. First, two independent reviewers screened the title and abstracts of the records for eligibility. In cases where an abstract was unavailable, records were preserved for a comprehensive full-text review. Second, the same investigators evaluated the complete text of potential studies to ascertain their final inclusion in the review. Finally, any discrepancies would be resolved by consensus with a senior reviewer.

### 2.4 Data Extraction

The following data from each included study were extracted by two independent reviewers: First author, publication year, country, source of study, design, sample size, participants characteristics (gender, age, type of stroke), exposure characteristics (type, timing), outcome characteristics (measurements, timing), missing data (loss rate, methods to missing data), and related methods of statistical analysis for association between BP and PSCI. If key information was missing from the study report, a communication would be dispatched to the original authors via email to acquire information. Any inconsistencies were addressed through discussions with a senior reviewer for resolution.

### 2.5 Quality Assessment

Two reviewers independently evaluated the risk of bias (RoB) for each study using modified iterations of the Newcastle–Ottawa scale [17]. The criteria for overall quality assessment were as follows [18]: (1) Good quality: Receiving 3 or 4 stars in the selection domain AND 1 or 2 stars in the comparability domain AND 2 or 3 stars in the outcome/exposure domain. (2) Fair quality: Receiving 2 stars in the selection domain AND 1 or 2 stars in the comparability domain AND 2 or 3 stars in the outcome/exposure domain. (3) Poor quality: Receiving 0 or 1 star in the selection domain OR 0 stars in the comparability domain OR 0 or 1 stars in the outcome/exposure domain.

### 2.6 Data Synthesis and Analysis

Meta-analyses were conducted utilizing RevMan 5.3 software (version 5.3, Cochrane Collaboration, Oxford, UK). The inverse variance method was used to pool effect estimates. Higgins *$I^{2}$* statistics was used to assess statistical heterogeneity. As described in Higgins’s study, we attributed low, moderate, and high descriptors to *$I^{2}$* values of 25%, 50%, and 75%, respectively [19]. If *$I^{2}$* ≥50%, a random effect model was applied in meta-analyses; otherwise, a fixed effect model was utilized. In consideration of inconsistency, subgroup analyses were conducted according to different levels of BP. If different levels of BP were reported in one study, we pooled these statics in the same or different subgroup analysis. Sensitivity analysis used the leave-one-out method to assess the robustness of pooled effect estimates. Forest plots were used for graphical representation. If sufficient studies (≥10) were included in the same outcome, we assessed publication bias using a funnel plot. Dichotomous data were provided as the OR and 95% confidence interval (CI). *p*-values equal to or below 0.05 were deemed statistically significant.

## 3. Results

### 3.1 Study Selection and Characteristics

**Fig. 1.**
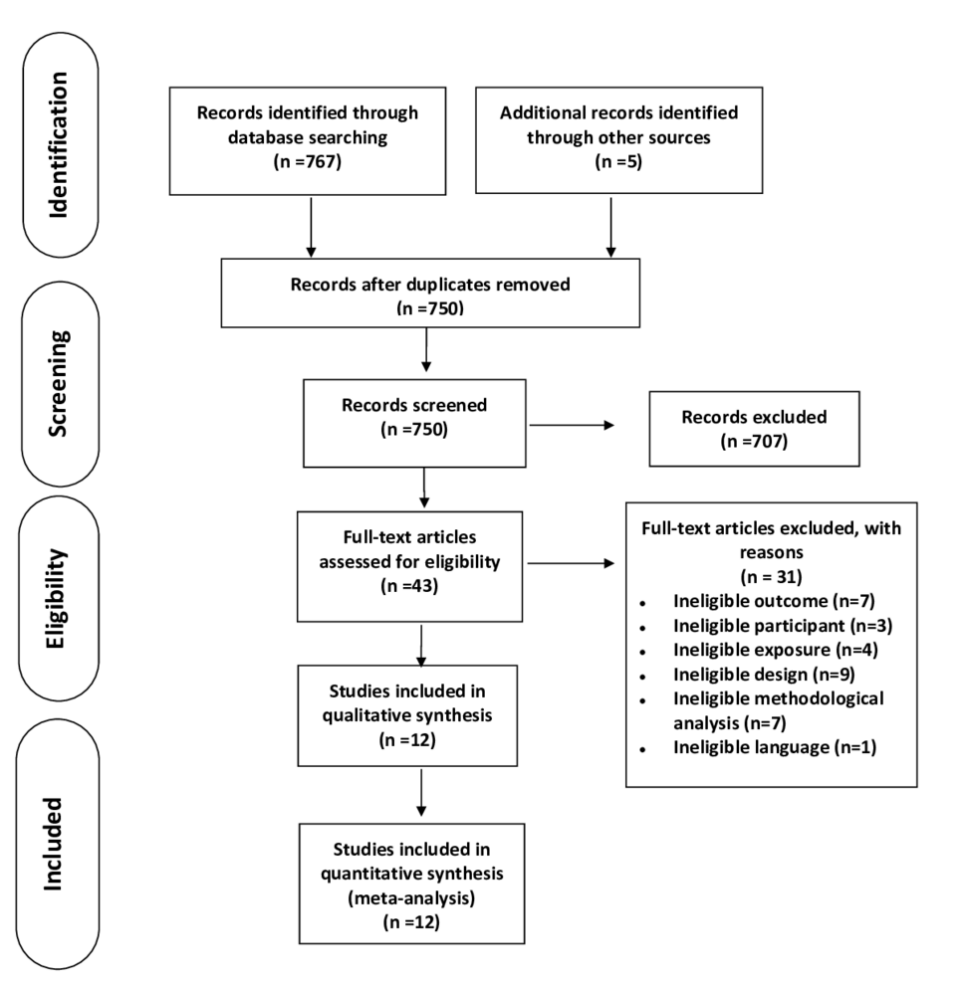
**Flow chart depicting the search procedure**. Systematic search of PubMed, the Cochrane Library, Embase, and Scopus was performed and identified 767 articles, with 5 additional articles identified through reference screening. After duplicate removal and title/abstract review, 43 citations were selected for full-text review. A total of 12 studies were included in the meta-analysis, with a total population of 21,732 patients.

A total of 767 articles were identified in the electronic databases (212 from PubMed, 191 from the Cochrane Library, 200 from Embase, and 164 from Scopus), with five additional articles identified through reference screening. After excluding 22 duplicates, 750 titles, and abstracts were reviewed, and 43 full-text articles were assessed. Finally, after excluding 31 studies for ineligible outcomes [20, 21, 22, 23, 24, 25, 26], participants [27, 28, 29], exposures [13, 30, 31, 32], study designs [33, 34, 35, 36, 37, 38, 39, 40], methodological analyses [4, 14, 15, 41, 42, 43], and published languages [44], 12 articles were identified as eligible and included in this meta-analysis. Fig. 1 illustrates the study selection process flow, including the reasons for exclusion.

Altogether, 12 articles [8, 11, 45, 46, 47, 48, 49, 50, 51, 52, 53, 54] with 21,732 participants were included (Table 1, Ref. [8, 11, 45, 46, 47, 48, 49, 50, 51, 52, 53, 54]). For different metrics of BP, six studies reported hypertension [46, 49, 51, 52, 53, 54], 3 reported SBP [47, 48, 50], and 1 reported DBP [45]. Individuals were classified as hypertensive if their BP exceeded 140/90 mmHg. Some of the included studies also reported the effects of different BP grades on PSCI [8, 11, 46, 47]. The details of BP measurements are presented in Table 2 (Ref. [8, 11, 45, 46, 47, 48, 49, 50, 51, 52, 53, 54]). For the timing of BP recorded, except for 1 study, which measured BP at the follow-up visit [46], the other 11 studies measured BP at baseline or admission [8, 11, 45, 47, 48, 49, 50, 51, 52, 53, 54]. For the tools used to measure PSCI, 11 studies used scales or examinations to test PSCI, and only 1 study reported PSCI by clinical diagnosis [46]. For the timing of PSCI being observed, the most common follow-up duration was 3 months (reported in 5 studies), the shortest was at discharge, and the longest was 4.4 years. Of these, 12 included studies [8, 11, 45, 46, 47, 48, 49, 50, 51, 52, 53, 54], 3 were secondary or post-hoc analyses from RCTs [8, 46, 48], 9 were observational studies [11, 45, 47, 49, 50, 51, 52, 53, 54], and every study used logistic regression for statistical analysis to explore the association between BP and PSCI.

**Table 1.** **Characteristics of included studies**.

Study	Country	Source of study	Design	Sample size (female, %)	Age group mean (SD)/median (range)	Type of stroke	Type of BP	Outcome measure tool	Outcome timing	Rate of missing data	ITT	Statistical analysis	
Mok *et al*. 2012 [45]	HK, China	VITATOPS	Prospective	100 (48)	75.17 (7.60)	Ischemic stroke	Baseline DBP	CDR	2 years	16%	Yes	Logistic regression	OR
Hilkens *et al*. 2021 [46]	Netherlands	PRoFESS	Post-hoc analysis from RCT	817 (41)	70.1 (8.5)	Non-cardioembolic ischemic stroke	Mean SBP	Clinical diagnosis at the final follow-up visit	Median 2.4 years (range 0–4.4 years)	1.2%	No	Logistic regression	OR
							Mean DBP						
							Hypertension						
Tuttolomondo *et al*. 2013 [47]	Italy	GIFA	Prospective	514 (52.91)	73.452 (6.2)	Acute ischemic stroke	SBP at admission	HAMT	Discharge	53.1%	No	Logistic regression	OR
You *et al*. 2017 [48]	Australia	INTERACT	Secondary analysis from RCT	231 (35.5)	62.3 (12.4)	Acute intracerebral hemorrhage	Mean SBP at baseline	MMSE	3 months	42.8%	No	Logistic regression	OR
Ihle-Hansen *et al*. 2015 [8]	Norway	NCT00506818	Secondary analysis from RCT	150 (44%)	71.5 (12.4)	Stroke	Baseline SBP	Clock Drawing Test	1 year	13%	No	Logistic regression	OR
Geng *et al*. 2017 [49]	China	ChiCTR-TRC-14004804	Prospective cohort	708 (45.9)	63.1 (10.0)	Acute ischemic stroke	Hypertension at admission	MoCA	3 months	11%	No	Logistic regression	OR
He *et al*. 2018 [11]	China	ChiCTR-TRC-14004804	Prospective cohort	796 (47)	63.19 (9.13)	Acute ischemic stroke	Mean SBP and DBP at baseline	MoCA	3 months	11%	No	Logistic regression	OR
Gong *et al*. 2020 [50]	China	NR	Prospective cohort	90 (35.5)	68.70 (12.76)	Intracerebral hemorrhage	SBP at admission	MoCA	2 weeks	NR	NA	Logistic regression	OR
Sarfo *et al*. 2017 [51]	Ghana	NR	Cross-sectional study	147 (47.6)	59.9 (13.7)	Stroke	Baseline hypertension	V-NB	NR	26.5%	No	Logistic regression	OR
Jacquin *et al*. 2014 [52]	France	NR	Prospective cohort	220 (44.1)	66.1 (16.6)	Stroke	Baseline hypertension	MMSE	3 months	21.4%	No	Logistic regression	OR
								MoCA					
Arba *et al*. 2017 [53]	UK	VISTA	Retrospective	1294 (35)	63.7 (11.8)	Stroke	Baseline hypertension	MMSE	1 year and 3 years	58.4% and 76.3%	No	Logistic regression	OR
Lu *et al*. 2019 [54]	China	NR	Prospective	213 (35)	64.8 (11.5)	Acute ischemic stroke	Baseline hypertension	MoCA	3 months	8.2%	No	Logistic regression	OR

Note: BP, blood pressure; ITT, intention-to-treat analysis; OR, odds ratio; VITATOPS, VITAmins TO Prevent Stroke study (NCT00097669); SBP, systolic blood pressure; DBP, diastolic blood pressure; CDR, Clinical Dementia Rating scale; MMSE, Mini-Mental State Examination; MoCA, Montreal Cognitive Assessment; PRoFESS, Prevention Regimen for Effectively avoiding Second Strokes (NCT00153062); GIFA, Gruppo Italiano di Farmacoepidemiologia nell’Anziano; HAMT, Hodkinson Abbreviated Mental Test; INTERACT, Intensive Blood Pressure Reduction in Acute Cerebral Hemorrhage Trial (NCT00226096); NR, not reported; V-NB, Vascular Neuropsychological Battery; VISTA, Virtual International Stroke Trial Archive; RCT, randomized controlled trial.

**Table 2.** **Details of BP measurements**.

Study	Hypertension	SBP	DBP	SBP grading (mmHg)	DBP grading (mmHg)
Mok *et al*. 2012 [45]			√		
Hilkens *et al*. 2021 [46]	√			<120	<70
				120–129	70–79
				130–139	80–89
				140–149	90–99
				150–159	≥100
				≥160	
Tuttolomondo *et al*. 2013 [47]		√		<120	
				>180	
You *et al*. 2017 [48]		√			
Ihle-Hansen *et al*. 2015 [8]				≤125	
				≤140	
				≤160	
Geng *et al*. 2017 [49]	√				
He *et al*. 2018 [11]				102–127	66–82
				128–142	83–92
				143–158	93–102
				159–170	103–109
				171–215	110–138
Gong *et al*. 2020 [50]		√			
Sarfo *et al*. 2017 [51]	√				
Jacquin *et al*. 2014 [52]	√				
Arba *et al*. 2017 [53]	√				
Lu *et al*. 2019 [54]	√				

Note: SBP, systolic blood pressure; DBP, diastolic blood pressure; BP, blood pressure; √, items that satisfied.

### 3.2 Quality Assessment

The general quality was assessed as good in five studies, fair in one study, and poor in six. In the included studies, BP was evaluated using dependable methods. For comparability, we considered baseline cognitive function or the National Institutes of Health Stroke Scale (NIHSS) score as key confounders and age, sex, antihypertensive treatment, and education as additional confounders. In the outcome assessment, four studies that reported PSCI were assessed by an independent, experienced neurologist or physician, while the other eight did not. The details of the RoB assessment are presented in Tables 3-1 (Ref. [8, 11, 45, 46, 47, 48, 49, 50, 52, 53, 54]) and 3-2 (Ref. [51]).

**Table 3-1.** **Adjudication of the quality of longitudinal studies included in the primary analysis**.

Study	Selection				Comparability		Outcome			Total
	Cohort representative	Non-exposed cohort	Exposure ascertainment	Outcome not presented at baseline	Controls for key confounder	Controls for additional confounder	Outcome assessment	Follow-up length	Follow-up rate	Overall quality
Mok *et al*. 2012 [45]	★	★	★	-	-	★	★	★	★	Good (8)
Hilkens *et al*. 2021 [46]	★	★	★	-	★	★	-	★	★	Good (7)
Tuttolomondo *et al*. 2013 [47]	★	★	★	-	-	-	-	-	-	Poor (3)
You *et al*. 2017 [48]	★	★	★	-	-	-	-	★	-	Poor (4)
Ihle-Hansen *et al*. 2015 [8]	★	★	★	-	★	★	-	★	★	Good (7)
Geng *et al*. 2017 [49]	★	★	★	-	★	★	-	★	★	Good (7)
He *et al*. 2018 [11]	★	★	★	-	★	★	★	★	★	Good (8)
Gong *et al*. 2020 [50]	★	★	★	-	-	★	-	-	-	Poor (4)
Jacquin *et al*. 2014 [52]	★	★	★	-	-	-	★	★	★	Poor (6)
Arba *et al*. 2017 [53]	★	★	★	-	★	★	-	★	-	Poor (6)
Lu *et al*. 2019 [54]	★	★	★	-	-	★	-	-	★	Poor (6)

Note: list of items and acceptable assessment (maximum stars per item): ∙ Selection (maximum 4 stars): 1. Representativeness of the exposed cohort: whether it is genuinely (all subjects or random sampling) or somewhat (non-random sampling) reflective of the average in the target population (★). 2. Selection of the non-exposed cohort: drawn from the identical community as the exposed cohort (★). 3. Ascertainment of the exposure (BP): hypertension, SBP, or DBP at baseline or follow-up visit (★). 4. Confirmation that the outcome of interest was absent at the commencement of the study: yes (★). ∙ Comparability (maximum 2 stars): study controls for the most important factor—baseline cognitive function or NIHSS score (★) and any additional factor or secondary important factor (age, sex, antihypertensive treatment, or education) (★). ∙ Outcome (maximum 3 stars): 1. Assessment of the outcome (PSCI or post-stroke dementia): objective and unbiased blind evaluation of PSCI or medical record linkage (★). 2. Was the follow-up duration sufficient to observe the occurrence of outcomes: most common—3 months from baseline to final cognitive assessment (★). 3. Adequacy of follow-up: either complete (with all subjects accounted for) or subjects lost to follow-up deemed unlikely to introduce bias, or a small number lost (<20%, or a detailed description of those lost) (★). Overall quality assessment (maximum 9 stars): ∙ Good quality: 3 or 4 stars in the selection domain AND 1 or 2 stars in the comparability domain AND 2 or 3 stars in the outcome domain. ∙ Fair quality: 2 stars in the selection domain AND 1 or 2 stars in the comparability domain AND 2 or 3 stars in the outcome domain. ∙ Poor quality: 0 or 1 star in the selection domain OR 0 stars in the comparability domain OR 0 or 1 stars in the outcome domain. BP, blood pressure; SBP, systolic blood pressure; DBP, diastolic blood pressure; PSCI, post-stroke cognitive impairment.

**Table 3-2.** **Adjudication of study quality for cross-sectional studies included in the meta-analysis**.

Study	Selection				Comparability		Outcome		Total
	Sample representative	Sample size justified/satisfactory	Non-respondents	Exposure ascertainment	Controls for key confounder	Controls for additional confounders	Outcome assessment	Statistical testing appropriate	Overall quality
Sarfo *et al*. 2017 [51]	★	-	-	★	-	★	★	★	Fair (5)

Note: list of items and acceptable assessment (maximum stars per item): ∙ Selection (maximum 4 stars): 1. Sample representative: truly (all subjects or random sampling) or somewhat (non-random sampling) reflective of the average in the target population (★). 2. Sample size: justified and satisfactory (★). 3. Non-respondents: equivalence in characteristics between respondents and non-respondents was confirmed, and the response rate is deemed satisfactory (★). 4. Ascertainment of the exposure (BP): hypertension, SBP, or DBP at baseline or follow-up visit (★). ∙ Comparability (maximum 2 stars): 1. Subjects in different outcome groups are comparable, and confounders are controlled: study controls for the most important factor—baseline cognitive function or NIHSS score (★) and any additional factor or secondary important factor (age, sex, antihypertensive treatment, or education) (★). ∙ Outcome (maximum 2 stars): 1. Assessment of the outcome (PSCI or post-stroke dementia): independent blind assessment of PSCI or medical record linkage (★). 2. Statistical test: test to describe the data clearly and appropriate measurement of the association is presented, including confidence intervals and *p*-value (★). Overall quality assessment (maximum 8 stars): ∙ Good quality: 3 or 4 stars in selection domain AND 1 or 2 stars in comparability domain AND 1 or 2 stars in outcome/exposure domain. ∙ Fair quality: 2 stars in the selection domain AND 1 or 2 stars in the comparability domain AND 1 or 2 stars in the outcome/exposure domain. ∙ Poor quality: 0 or 1 star in the selection domain OR 0 stars in the comparability domain OR 0 or 1 stars in the outcome/exposure domain. BP, blood pressure; SBP, systolic blood pressure; DBP, diastolic blood pressure; PSCI, post-stroke cognitive impairment.

### 3.3 Hypertension and PSCI 

**Fig. 2.**
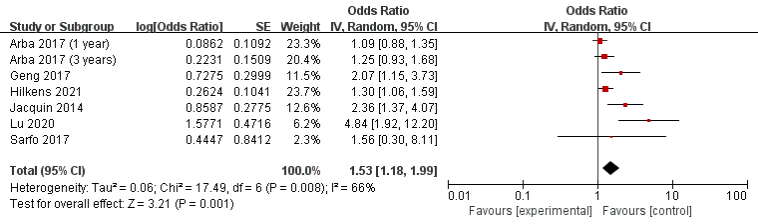
**Forest plot of the association between hypertension and post-stroke cognitive impairment (PSCI)**. SE, standard error; CI, confidence interval; *p*-value of 0.05 or less indicated statistical significance.

A total of six studies were incorporated into the meta-analysis in terms of hypertension and PSCI. A forest plot (Fig. 2) showed that hypertension significantly increased the odds of PSCI in patients following a stroke (OR 1.53, 95% CI, 1.18–1.99; *p* = 0.001). The statistical heterogeneity was moderate (*$I^{2}$* = 66%).

### 3.4 SBP and PSCI

**Fig. 3.**
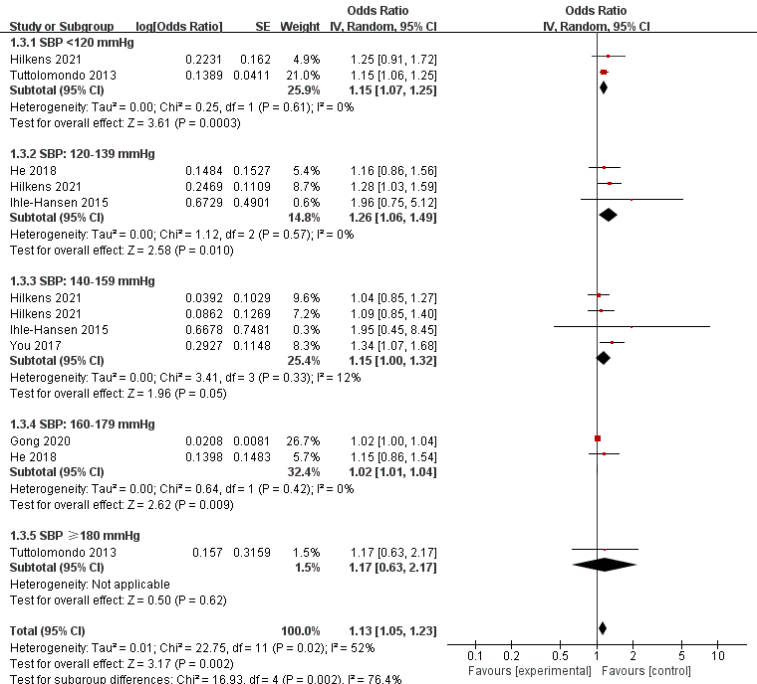
**Forest plot of association between SBP and PSCI**. SE, standard error; CI, confidence interval; *p*-value of 0.05 or less indicated statistical significance. SBP, systolic blood pressure; PSCI, post-stroke cognitive impairment.

The meta-analysis included 12 SBPs. Forest plot (Fig. 3) showed that SBP was significantly associated with the incidence of PSCI (OR 1.13, 95% CI, 1.05–1.23; *p* = 0.002). Furthermore, people with an SBP <120 mmHg, 120–139 mmHg, 140–159 mmHg, or 160–179 mmHg were more likely to have PSCI (OR 1.15, 95% CI, 1.07–1.25; *p* = 0.0003; OR 1.26, 95% CI, 1.06–1.49; *p* = 0.010; OR 1.15, 95% CI, 1.00–1.32; *p* = 0.05; OR 1.02, 95% CI, 1.01–1.04; *p* = 0.009, respectively). However, when SBP ≥180 mmHg, the association between SBP and PSCI became non-significant (OR 1.17, 95% CI, 0.63–2.17; *p* = 0.62). The overall statistical heterogeneity was moderate (*$I^{2}$* = 52%).

### 3.5 DBP and PSCI

**Fig. 4.**
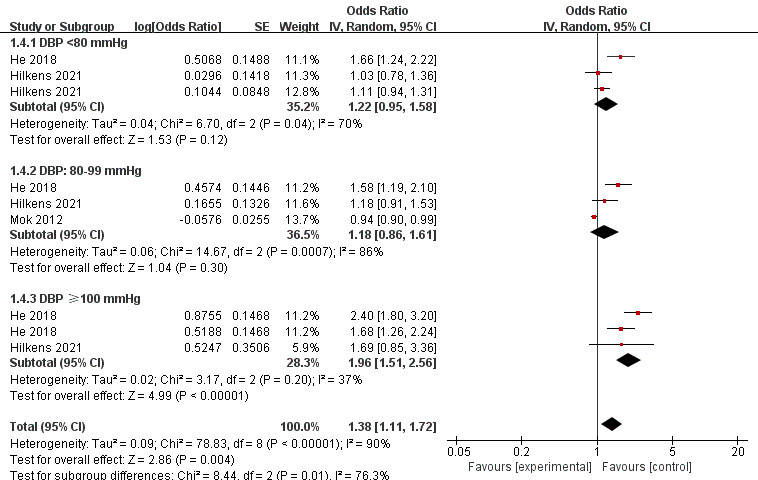
**Forest plot of association between DBP and PSCI**. SE, standard error; CI, confidence interval; *p*-value of 0.05 or less indicated statistical significance. DBP, diastolic blood pressure; PSCI, post-stroke cognitive impairment.

The meta-analysis included nine DBPs. The forest plot (Fig. 4) showed that the DBP could significantly predict the odds of PSCI (OR 1.38, 95% CI, 1.11–1.72; *p* = 0.004). In the subgroup analysis, DBP <80 mmHg and 80–99 mmHg did not significantly increase the odds of PSCI (OR 1.22, 95% CI, 0.95–1.58; *p* = 0.12; OR 1.18, 95% CI, 0.86–1.61; *p* = 0.30, respectively). While for people with DBP ≥100 mmHg, the incidence of PSCI was significantly increased (OR 1.96, 95% CI, 1.51–2.56; *p* < 0.00001). The overall statistical heterogeneity was high (*$I^{2}$* = 90%).

### 3.6 Sensitivity Analysis

The sensitivity analysis did not affect the pooled effect estimates; the overall effect remained significant when we removed the studies one by one. Furthermore, when only studies on ischemic stroke were retained in this meta-analysis, the pooled effect estimates remained unaffected. Considering that only Hilkens *et al*. [46] measured BP at follow-ups, we removed this study from the meta-analysis to detect the robustness of pooled effect estimates, which remained significant, thereby indicating that the meta-analysis results were robust. Nevertheless, when we removed Gong’s study for the SBP and PSCI outcomes, the overall heterogeneity changed from 52% to 0%, meaning Gong’s study could be the main source of the moderate heterogeneity.

### 3.7 Publication Bias

**Fig. 5.**
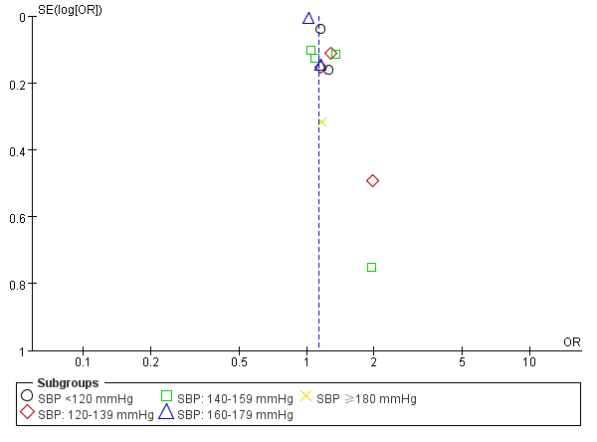
**Funnel plot of publication bias by SBP for PSCI**. OR, odds ratio; SE, standard error; SBP, systolic blood pressure; PSCI, post-stroke cognitive impairment..

More than 10 studies were incorporated into the SBP and PSCI outcomes meta-analysis. The funnel plot (Fig. 5) showed the existing obvious publication bias, which may be due to some negative results in the unpublished studies.

## 4. Discussion 

### 4.1 Main Findings

This meta-analysis showed that for post-stroke patients (aged from 46 to 83 years), hypertension, SBP, and DBP measured at baseline or follow-ups are risk factors for PSCI. This finding applies to common subtypes of stroke, such as ischemic and hemorrhagic stroke. Additionally, during the 4-year period after a stroke, the onset of cognitive impairment is closely related to BP, and this effect begins as early as being discharged. Furthermore, SBP or DBP at different intervals have different effects on the occurrence and progression of PSCI. The incidence of cognitive impairment in stroke patients with SBP ≥180 mmHg or DBP <100 mmHg may be less influenced by BP, while stroke patients with SBP <120 mmHg, 120–179 mmHg or DBP ≥100 mmHg are more likely to develop PSCI. However, due to limited included studies and statistical heterogeneity, the evidence of this meta-analysis may be low, and more research is needed to support our findings.

### 4.2 Comparison with Other Studies

A previous systematic review presented a qualitative analysis of the association between BP and cognitive impairment for non-stroke patients [55] and highlighted that people with hypertension tended to demonstrate a more pronounced decline in global cognitive functioning compared to normotensive individuals. This finding strengthens the evidence of our work. Hypertension has been shown to be a risk factor for cognitive decline in patients with or without stroke [55, 56]. For patients with stroke, a previous study published in 2009 performed a meta-analysis of predictors for pre- and post-stroke dementia, and an insignificant association was found between hypertension and PSCI [5], contradicting the findings of this meta-analysis. A recent meta-analysis published in 2022 supported that hypertension was a predictor of post-stroke dementia [57]. The main sources of discrepancy are methodological differences and heterogeneity of included studies. More rigorous designed studies are required to detect the predictive significance of hypertension in PSCI.

In addition, our work also found SBP or DBP was significantly associated with an increase in PSCI, and this finding was similar to that of a previous meta-analysis [18], which supported that mean SBP or DBP was a risk factor for cognitive decline in patients without stroke. Furthermore, we found that both SBP <120 mmHg or 120–179 mmHg and DBP ≥100 mmHg were predictors of PSCI, which indicated that both normal and abnormal systolic or diastolic BP in the early post-stroke period might influence the development of PSCI; however, this effect may be indirect rather than independent or U-shaped according to previous studies [10, 11, 12]. Therefore, it is unclear whether early or long-term use of antihypertensive treatments after stroke can reduce the incidence of PSCI, while a previous study has also disputed the use of antihypertensive treatments after stroke to prevent PSCI [58]. In brief, SBP or DBP at different intervals significantly increases the incidence of PSCI, although whether this relationship is U-shaped or direct remains to be debated.

### 4.3 Advantages and Limitations of the Study

The current meta-analysis exhibits various strengths and limitations that warrant acknowledgment. The strengths include the following: First, this was the first meta-analysis that provided evidence of hypertension, SBP, and DBP on PSCI, and a significant association was found between BP and PSCI. Second, we conducted subgroup analysis according to different cut-off values of BP and found that BP at different intervals had different predictive effects of PSCI. These findings provide more theoretical and practical significance for future research. Third, this meta-analysis was performed according to MOOSE report guidelines [16]. Systematic reviews of observational studies were considered to possess significant limitations and methodological intricacies attributed to the observational nature of the studies from which they are derived [59]. Therefore, the MOOSE checklist was selected for this meta-analysis to strengthen the evidence.

As a limitation: First of all, caution should be exercised in interpreting the results of this meta-analysis owing to the restricted number of studies included and its moderate to high statistical heterogeneity. This meta-analysis included different study designs, such as observational studies and secondary analyses from RCTs. Therefore, heterogeneity arising from different study designs tends to be unavoidable, which is similar to previous meta-analyses [56]. This meta-analysis used a random effect model to minimize potential heterogeneity. In addition, heterogeneity between studies (measurement methods and data reporting) is common in observational studies; precisely, if all original studies were from the same study without heterogeneity, the larger the overall estimated variance, the larger the sample size required. Generic inverse variance is a weighting method based on the variance of the effect, reflecting the estimated effect of a pooled sample. Therefore, according to previous studies [56, 60], we used a random-effects model with a generic inverse variance method of log (OR) to conduct a meta-analysis. Furthermore, different outcome measurement tools could explain measurement bias across studies, although these are global standardized tools to define PSCI [1]. Currently, there is no definite recommendation about the sensitivity of different tools measuring PSCI; thus, for future studies, we could include specific tools to measure PSCI to reduce measurement bias.

### 4.4 Implications for Research

At first, we found a significant but not U-shaped relationship between BP and PSCI. Subsequent research is required to investigate the association between BP at different intervals and PSCI. Second, high BP impacts cerebral perfusion, inducing adaptive vascular alterations, and these changes render the brain susceptible, potentially predisposing it to the onset of cognitive impairment [61, 62]. However, the etiology of PSCI is complicated; more studies are needed to explore the mechanism of high BP on PSCI. Lastly, multiple co-variables take effect when performing a regression analysis of BP on PSCI, and previous studies reported that baseline cognitive function was also a significant predictor for PSCI [58]. However, few of the included studies adjusted baseline cognitive function; thus, future studies need to take more into account when exploring predictors of PSCI, and multi-level meta-regression analysis is needed in the future to present more important predictors of PSCI.

### 4.5 Implication for Clinical Practice

Through meta-analysis, a notable correlation was identified between hypertension, abnormal or normal BP, and PSCI. Therefore, in individuals who have previously experienced hypertension, the use of antihypertensive treatments can prevent PSCI. However, for non-hypertensive patients with abnormal or normal BP in the early stages post-stroke, the use of antihypertensive treatments may have little significance in preventing PSCI. Additionally, considering the predictive value of BP on PSCI, regular and long-term follow-ups for BP should be performed in patients following a stroke. Finally, the etiology of PSCI is individualized, meaning PSCI is not only affected by BP, therefore, clinicians should consider all important factors of patients in order to make correct decisions on treatment.

## 5. Conclusions

Hypertension, SBP, or DBP has predictive values for PSCI, and SBP <120 mmHg, 120–179 mmHg, or DBP ≥100 mmHg has different significant associations with PSCI. However, more studies are needed in the future to detect the relationship between BP and PSCI. Considering the overall quality of this meta-analysis to be low, these results should be interpreted with caution.

## Data Availability

The datasets generated during and/or analyzed during the current study are available from the corresponding author on reasonable request.

## Supplementary Material

Supplementary material associated with this article can be found, in the online version, at https://doi.org/10.31083/j.rcm2505174.

## References

[1] Rost NS, Brodtmann A, Pase MP, van Veluw SJ, Biffi A, Duering M (2022). Post-Stroke Cognitive Impairment and Dementia. *Circulation Research*.

[2] Lo JW, Crawford JD, Desmond DW, Bae HJ, Lim JS, Godefroy O (2022). Long-Term Cognitive Decline After Stroke: An Individual Participant Data Meta-Analysis. *Stroke*.

[3] Sun JH, Tan L, Yu JT (2014). Post-stroke cognitive impairment: epidemiology, mechanisms and management. *Annals of Translational Medicine*.

[4] Levine DA, Wadley VG, Langa KM, Unverzagt FW, Kabeto MU, Giordani B (2018). Risk Factors for Poststroke Cognitive Decline: The REGARDS Study (Reasons for Geographic and Racial Differences in Stroke). *Stroke*.

[5] Pendlebury ST, Rothwell PM (2009). Prevalence, incidence, and factors associated with pre-stroke and post-stroke dementia: a systematic review and meta-analysis. *The Lancet. Neurology*.

[6] Georgakis MK, Duering M, Wardlaw JM, Dichgans M (2019). WMH and long-term outcomes in ischemic stroke: A systematic review and meta-analysis. *Neurology*.

[7] Willmot M, Leonardi-Bee J, Bath PMW (2004). High blood pressure in acute stroke and subsequent outcome: a systematic review. *Hypertension (Dallas, Tex.: 1979)*.

[8] Ihle-Hansen H, Thommessen B, Fagerland MW, Øksengård AR, Wyller TB, Engedal K (2015). Blood pressure control to prevent decline in cognition after stroke. *Vascular Health and Risk Management*.

[9] Moretti R, Torre P, Antonello RM, Manganaro D, Vilotti C, Pizzolato G (2008). Risk factors for vascular dementia: hypotension as a key point. *Vascular Health and Risk Management*.

[10] Goodfellow JA, Dawson J, Quinn TJ (2013). Management of blood pressure in acute stroke. *Expert Review of Neurotherapeutics*.

[11] He M, Wang J, Liu N, Xiao X, Geng S, Meng P (2018). Effects of Blood Pressure in the Early Phase of Ischemic Stroke and Stroke Subtype on Poststroke Cognitive Impairment. *Stroke*.

[12] Boreas AM, Lodder J, Kessels F, de Leeuw PW, Troost J (2001). Predictors of poststroke blood pressure level and course. *Journal of Stroke and Cerebrovascular Diseases: the Official Journal of National Stroke Association*.

[13] Bu X, Zhang Y, Bazzano LA, Xu T, Guo L, Wang X (2016). Effects of early blood pressure reduction on cognitive function in patients with acute ischemic stroke. *International Journal of Stroke: Official Journal of the International Stroke Society*.

[14] Kim Y, Lim JS, Oh MS, Yu KH, Lee JS, Park JH (2021). Blood pressure variability is related to faster cognitive decline in ischemic stroke patients: PICASSO subanalysis. *Scientific Reports*.

[15] Levine DA, Galecki AT, Okullo D, Briceño EM, Kabeto MU, Morgenstern LB (2020). Association of Blood Pressure and Cognition after Stroke. *Journal of Stroke and Cerebrovascular Diseases: the Official Journal of National Stroke Association*.

[16] Brooke BS, Schwartz TA, Pawlik TM (2021). MOOSE Reporting Guidelines for Meta-analyses of Observational Studies. *JAMA Surgery*.

[17] Wells G, Shea B, O’Connell D, Peterson J, Welch V, Losos M (2023). The newcastle-ottawa scale (nos) for assessing the quality of nonrandomised studies in meta-analyses. The Ottawa Hospital. https://www.ohri.ca/programs/clinical_epidemiology/oxford.asp.

[18] de Heus RAA, Tzourio C, Lee EJL, Opozda M, Vincent AD, Anstey KJ (2021). Association Between Blood Pressure Variability With Dementia and Cognitive Impairment: A Systematic Review and Meta-Analysis. *Hypertension (Dallas, Tex.: 1979)*.

[19] Higgins JPT, Thompson SG, Deeks JJ, Altman DG (2003). Measuring inconsistency in meta-analyses. *BMJ (Clinical Research Ed.)*.

[20] de Havenon A, Bennett A, Stoddard GJ, Smith G, Wang H, Wold J (2016). Increased Blood Pressure Variability Is Associated with Worse Neurologic Outcome in Acute Anterior Circulation Ischemic Stroke. *Stroke Research and Treatment*.

[21] Viswanathan A, Guichard JP, Gschwendtner A, Buffon F, Cumurcuic R, Boutron C (2006). Blood pressure and haemoglobin A1c are associated with microhaemorrhage in CADASIL: a two-centre cohort study. *Brain: a Journal of Neurology*.

[22] Kobayashi J, Koga M, Tanaka E, Okada Y, Kimura K, Yamagami H (2014). Continuous antihypertensive therapy throughout the initial 24 hours of intracerebral hemorrhage: the stroke acute management with urgent risk-factor assessment and improvement-intracerebral hemorrhage study. *Stroke*.

[23] Erlebach R, Bächli E, Gerrits E, Hess M (2021). Stroke management in a Swiss community hospital - in close collaboration with a stroke centre. *Swiss Medical Weekly*.

[24] Tennen G, Herrmann N, Black SE, Levy KS, Cappell J, Li A (2011). Are vascular risk factors associated with post-stroke depressive symptoms. *Journal of Geriatric Psychiatry and Neurology*.

[25] Vaughan L, Bushnell C, Bell CL, Espeland MA (2016). Global cognitive function before, surrounding, and after ischemic stroke: the role of risk and protective factors varies with time among ischemic stroke survivors. *Neuropsychology, Development, and Cognition. Section B, Aging, Neuropsychology and Cognition*.

[26] Ningning W, Ying H, Shudong L, Zhilong Z, Qibo C, Yuting D (2022). Blood pressure variability related to early outcome of acute ischemia stroke in a prospective observational study. *Medicine*.

[27] Zhou TL, Kroon AA, van Sloten TT, van Boxtel MPJ, Verhey FRJ, Schram MT (2019). Greater Blood Pressure Variability Is Associated With Lower Cognitive Performance. *Hypertension (Dallas, Tex.: 1979)*.

[28] Zhang J, Liu L, Sun H, Li M, Li Y, Zhao J (2018). Cerebral Microbleeds Are Associated With Mild Cognitive Impairment in Patients With Hypertension. *Journal of the American Heart Association*.

[29] Shlyakhto E (2007). Observational Study on Cognitive function And systolic blood pressure Reduction (OSCAR): preliminary analysis of 6-month data from > 10,000 patients and review of the literature. *Current Medical Research and Opinion*.

[30] Sachdev PS, Brodaty H, Valenzuela MJ, Lorentz L, Looi JCL, Berman K (2006). Clinical determinants of dementia and mild cognitive impairment following ischaemic stroke: the Sydney Stroke Study. *Dementia and Geriatric Cognitive Disorders*.

[31] Wang Z, Wong A, Liu W, Yang J, Chu WCW, Au L (2015). Pulse Pressure and Cognitive Decline in Stroke Patients With White Matter Changes. *Journal of Clinical Hypertension (Greenwich, Conn.)*.

[32] Al Fawal B, Ibrahim A, Abd Elhamed M (2021). Post-stroke dementia: frequency, predictors, and health impact. *Egyptian Journal of Neurology, Psychiatry and Neurosurgery*.

[33] Benavente OR, White CL, Pearce L, Pergola P, Roldan A, Benavente MF (2011). The Secondary Prevention of Small Subcortical Strokes (SPS3) study. *International Journal of Stroke: Official Journal of the International Stroke Society*.

[34] Wardlaw JM, Doubal F, Brown R, Backhouse E, Woodhouse L, Bath P (2021). Rates, risks and routes to reduce vascular dementia (R4vad), a UK-wide multicentre prospective observational cohort study of cognition after stroke: Protocol. *European Stroke Journal*.

[35] Chatterjee A (2016). Cognitive outcome of stroke patients. *International Journal of Stroke*.

[36] Aronow WS (2017). Hypertension and cognitive impairment. *Annals of Translational Medicine*.

[37] Gong J, Harris K, Tzourio C, Harrap S, Naismith S, Anderson CS (2021). Sex differences in predictors for cognitive decline and dementia in people with stroke or transient ischemic attack in the PROGRESS trial. *International Journal of Stroke: Official Journal of the International Stroke Society*.

[38] Gorelick PB, Whelton PK, Sorond F, Carey RM (2020). Blood Pressure Management in Stroke. *Hypertension (Dallas, Tex.: 1979)*.

[39] Li S, Liu Q, Li Y, Hu Z, Jiang X, Wang X (2017). Characteristics and risk factors analysis of cognitive decline in elderly patients with cerebral infarction. *Chinese Journal of Cerebrovascular Diseases*.

[40] Hennerici MG (2009). What are the mechanisms for post-stroke dementia. *The Lancet. Neurology*.

[41] Levine DA, Haan MN, Langa KM, Morgenstern LB, Neuhaus J, Lee A (2013). Impact of gender and blood pressure on poststroke cognitive decline among older Latinos. *Journal of Stroke and Cerebrovascular Diseases: the Official Journal of National Stroke Association*.

[42] Yamamoto Y, Nagakane Y, Tomii Y, Akiguchi I (2016). High Morning and Bedtime Home Blood Pressures Strongly Predict for Post-Stroke Cognitive Impairment. *Journal of Stroke and Cerebrovascular Diseases: the Official Journal of National Stroke Association*.

[43] Lee JH, Oh E, Oh MS, Kim C, Jung S, Park JH (2014). Highly variable blood pressure as a predictor of poor cognitive outcome in patients with acute lacunar infarction. *Cognitive and Behavioral Neurology: Official Journal of the Society for Behavioral and Cognitive Neurology*.

[44] Widimský J (2003). Management of dementia and cognitive functions in patients after cerebrovascular stroke: new results from the PROGRESS study. *Vnitrni Lekarstvi*.

[45] Mok V, Xiong Y, Wong KK, Wong A, Schmidt R, Chu WWC (2012). Predictors for cognitive decline in patients with confluent white matter hyperintensities. *Alzheimer’s & Dementia: the Journal of the Alzheimer’s Association*.

[46] Hilkens NA, Klijn CJM, Richard E (2021). Blood pressure, blood pressure variability and the risk of poststroke dementia. *Journal of Hypertension*.

[47] Tuttolomondo A, Di Raimondo D, Di Sciacca R, Pedone C, La Placa S, Arnao V (2013). Effects of clinical and laboratory variables at admission and of in-hospital treatment with cardiovascular drugs on short term prognosis of ischemic stroke. The GIFA study. *Nutrition, Metabolism, and Cardiovascular Diseases: NMCD*.

[48] You S, Wang X, Lindley RI, Robinson T, Anderson CS, Cao Y (2017). Early Cognitive Impairment after Intracerebral Hemorrhage in the INTERACT1 Study. *Cerebrovascular Diseases (Basel, Switzerland)*.

[49] Geng S, Liu N, Meng P, Ji N, Sun Y, Xu Y (2017). Midterm Blood Pressure Variability Is Associated with Poststroke Cognitive Impairment: A Prospective Cohort Study. *Frontiers in Neurology*.

[50] Gong L, Gu Y, Yu Q, Wang H, Zhu X, Dong Q (2020). Prognostic Factors for Cognitive Recovery Beyond Early Poststroke Cognitive Impairment (PSCI): A Prospective Cohort Study of Spontaneous Intracerebral Hemorrhage. *Frontiers in Neurology*.

[51] Sarfo FS, Akassi J, Adamu S, Obese V, Ovbiagele B (2017). Burden and Predictors of Poststroke Cognitive Impairment in a Sample of Ghanaian Stroke Survivors. *Journal of Stroke and Cerebrovascular Diseases: the Official Journal of National Stroke Association*.

[52] Jacquin A, Binquet C, Rouaud O, Graule-Petot A, Daubail B, Osseby GV (2014). Post-stroke cognitive impairment: high prevalence and determining factors in a cohort of mild stroke. *Journal of Alzheimer’s Disease: JAD*.

[53] Arba F, Quinn T, Hankey GJ, Inzitari D, Ali M, Lees KR (2017). Determinants of post-stroke cognitive impairment: analysis from VISTA. *Acta Neurologica Scandinavica*.

[54] Lu ZH, Li J, Li XL, Ding M, Mao CJ, Zhu XY (2019). Hypertension with Hyperhomocysteinemia Increases the Risk of Early Cognitive Impairment after First-Ever Ischemic Stroke. *European Neurology*.

[55] Forte G, De Pascalis V, Favieri F, Casagrande M (2019). Effects of Blood Pressure on Cognitive Performance: A Systematic Review. *Journal of Clinical Medicine*.

[56] Ball EL, Shah M, Ross E, Sutherland R, Squires C, Mead GE (2023). Predictors of post-stroke cognitive impairment using acute structural MRI neuroimaging: A systematic review and meta-analysis. *International Journal of Stroke: Official Journal of the International Stroke Society*.

[57] Waziry R, Claus JJ, Hofman A (2022). Dementia Risk Following Ischemic Stroke: A Systematic Review and Meta-Analysis of Factors Collected at Time of Stroke Diagnosis. *Journal of Alzheimer’s Disease: JAD*.

[58] Mijajlović MD, Pavlović A, Brainin M, Heiss WD, Quinn TJ, Ihle-Hansen HB (2017). Post-stroke dementia - a comprehensive review. *BMC Medicine*.

[59] Vandenbroucke JP, von Elm E, Altman DG, Gøtzsche PC, Mulrow CD, Pocock SJ (2007). Strengthening the Reporting of Observational Studies in Epidemiology (STROBE): explanation and elaboration. *PLoS Medicine*.

[60] Yang Z, Wang H, Edwards D, Ding C, Yan L, Brayne C (2020). Association of blood lipids, atherosclerosis and statin use with dementia and cognitive impairment after stroke: A systematic review and meta-analysis. *Ageing Research Reviews*.

[61] Nasrabady SE, Rizvi B, Goldman JE, Brickman AM (2018). White matter changes in Alzheimer’s disease: a focus on myelin and oligodendrocytes. *Acta Neuropathologica Communications*.

[62] Mahoney CJ, Ridgway GR, Malone IB, Downey LE, Beck J, Kinnunen KM (2014). Profiles of white matter tract pathology in frontotemporal dementia. *Human Brain Mapping*.

