# Supplementary material for: Association between Blood Pressure and Post-Stroke Cognitive Impairment: A Meta-Analysis
Source: Rev Cardiovasc Med. 2024 May 16;25(5):174. doi: 10.31083/j.rcm2505174 (PMC11267189; doi:10.31083/j.rcm2505174)
Supplement: Supplementary file 1 [file 2153-8174-25-5-174-s1.zip › 2153-8174-25-5-174-s1/Supplementary Material.docx]

| Supplementary: Search key terms and strategy | | |
| --- | --- | --- |
| PubMed | | |
|  | Filters applied | Human subjects, adult subjects, language (English), multicenter study, observational study |
|  | Search | "Blood Pressure"[Title/Abstract] OR "blood pressure monitoring"[Title/Abstract] OR "systolic blood pressure"[Title/Abstract] OR "diastolic blood pressure"[Title/Abstract] OR "average blood pressure"[Title/Abstract] OR "mean arteria blood pressure"[Title/Abstract] OR "Blood Pressure"[MeSH Terms] OR "Hypotension"[MeSH Terms] OR "Hypertension"[MeSH Terms]  AND  "Cognition"[MeSH Terms] OR "Cognition Disorders"[MeSH Terms] OR "Mental Status and Dementia Tests"[MeSH Terms] OR "Cognitive Dysfunction"[MeSH Terms] OR "Neuropsychological Tests"[MeSH Terms] OR "Neurobehavioral Manifestations"[MeSH Terms] OR "Attention"[MeSH Terms] OR "Orientation"[MeSH Terms] OR "Memory"[MeSH Terms] OR "Apraxias"[MeSH Terms] OR "Executive Function"[MeSH Terms] OR "Processing Speed"[MeSH Terms] OR "Dementia"[MeSH Terms] OR "dementia, vascular"[MeSH Terms] OR "dementia, multi infarct"[MeSH Terms] OR "Mixed Dementias"[MeSH Terms] OR "Visuospatial ability"[Title/Abstract] OR "Cognitive impairment"[Title/Abstract] OR "Cognitive decline"[Title/Abstract] OR "Cognitive defect"[Title/Abstract] OR "cognitive assess*"[Title/Abstract] OR "cognitive test*"[Title/Abstract] OR "Mini Mental State"[Title/Abstract] OR "MoCA"[Title/Abstract] OR "mental deterioration"[Title/Abstract] OR "vascular dementia"[Title/Abstract]  AND  "stroke"[MeSH Terms] OR "stroke"[All Fields] OR ("vascular"[All Fields] AND "accident"[All Fields] AND "brain"[All Fields] OR "vascular accident, brain"[All Fields] OR ("cerebrovascular"[All Fields] AND "accident"[All Fields]) OR "cerebrovascular accident"[All Fields] OR "cerebrovascular disorders"[MeSH Terms] OR ("cerebrovascular"[All Fields] AND "disorders"[All Fields]) OR ("cerebrovascular"[All Fields] AND "disease"[All Fields]) OR "cerebrovascular disease"[All Fields] OR "brain ischaemia"[All Fields] OR "brain ischemia"[MeSH Terms] OR ("brain"[All Fields] AND "ischemia"[All Fields]) OR "brain ischemia"[All Fields] "transient ischaemic attack"[All Fields] OR "ischemic attack, transient"[MeSH Terms] OR ("ischemic"[All Fields] AND "attack"[All Fields] AND "transient"[All Fields]) OR "transient ischemic attack"[All Fields] "cerebral haemorrhage"[All Fields] OR "cerebral hemorrhage"[MeSH Terms] OR ("cerebral"[All Fields] AND "hemorrhage"[All Fields]) OR "Hemorrhagic Stroke"[MeSH Terms] OR "Embolic Stroke"[MeSH Terms] OR "Thrombotic Stroke"[MeSH Terms] OR "Ischemic Stroke"[MeSH Terms] OR "intracranial hemorrhage, hypertensive"[MeSH Terms] OR "Intracranial Hemorrhages"[MeSH Terms] |
| the Cochrane library | | |
| Filters applied | | Human subjects, adult subjects, language (English), multicenter study, observational study |
| Search | | #1 MeSH descriptor: [Blood Pressure] explode all trees  #2 MeSH descriptor: [Hypertension] explode all trees  #3 MeSH descriptor: [Hypotension] explode all trees  #4 ("blood pressure monitoring"):ti,ab,kw  #5 ("systolic blood pressure"):ti,ab,kw  #6 ("diastolic blood pressure"):ti,ab,kw  #7 ("average blood pressure"):ti,ab,kw  #8 #1 OR #2 OR #3 OR #4 OR #5 OR #6 OR #7  #9 MeSH descriptor: [Cognition] explode all trees  #10 MeSH descriptor: [Attention] explode all trees  #11 MeSH descriptor: [Orientation] explode all trees  #12 MeSH descriptor: [Memory] explode all trees  #13 MeSH descriptor: [Apraxias] explode all trees  #14 MeSH descriptor: [Executive Function] explode all trees  #15 MeSH descriptor: [Processing Speed] explode all trees  #16 MeSH descriptor: [Cognitive Dysfunction] explode all trees  #17 MeSH descriptor: [Mental Status and Dementia Tests] explode all trees  #18 MeSH descriptor: [Neuropsychological Tests] explode all trees  #19 MeSH descriptor: [Dementia] explode all trees  #20 ("cognitive impairment"):ti,ab,kw  #21 ("cognitive decline"):ti,ab,kw  #22 ("cognitive defect"):ti,ab,kw  #23 ("vascular dementia"):ti,ab,kw  #24 #9 OR #10 OR #11 OR #12 OR #13 OR #14 OR #15 OR #16 OR #17 OR #18 OR #19 OR #20 OR #21 OR #22 OR #23  #25 MeSH descriptor: [Stroke] explode all trees  #26 MeSH descriptor: [Cerebral Hemorrhage] explode all trees  #27 MeSH descriptor: [Ischemic Attack, Transient] explode all trees  #28 MeSH descriptor: [Cerebral Small Vessel Diseases] explode all trees  #29 MeSH descriptor: [Brain Ischemia] explode all trees  #30 MeSH descriptor: [Cerebrovascular Disorders] explode all trees  #31 MeSH descriptor: [Intracranial Embolism] explode all trees  #32 MeSH descriptor: [Intracranial Hemorrhages] explode all trees  #33 (apoplexy):ti,ab,kw  #34 "cerebral hemorrhage"  #35 "cerebral infarct"  #36 "brain infarct"  #37 "transient ischemic attack"  #38 "cerebral vascular disorder"  #39 "Cerebrovascular Accident"  #40 "Brain Vascular Accident"  #41 #25 OR #26 OR #27 OR #28 OR #29 OR #30 OR #31 OR #32 OR #33 OR #34 OR #35 OR #36 OR #37 OR #38 OR #39 OR #40  #42 #8 AND #24 AND #41 |
| Embase | | |
|  | Filters applied | Human subjects, adult subjects, language (English), multicenter study, observational study |
|  | Search | 'blood pressure'/exp OR 'blood pressure monitoring'/exp OR 'systolic blood pressure'/exp OR 'diastolic blood pressure'/exp OR 'elevated blood pressure'/exp OR 'hypertension'/exp OR 'depressed blood pressure'/exp OR 'hypotension'/exp OR 'ambulatory blood pressure':ti,ab,kw OR 'average blood pressure':ti,ab,kw OR 'mean arteria blood pressure':ti,ab,kw  AND  'cognition'/exp OR 'cognitive defect'/exp OR 'cognitive function test'/exp OR 'neuropsychological assessment'/exp OR 'attention test'/exp OR 'executive function test'/exp OR 'memory test'/exp OR 'visual-spatial ability test'/exp OR 'orientation'/exp OR 'apraxia'/exp OR 'processing speed'/exp OR 'dementia'/exp OR 'mental deterioration'/exp OR 'multiinfarct dementia'/exp OR 'mixed dementia'/exp OR 'cognitive impairment':ti,ab,kw OR 'cognitive decline':ti,ab,kw OR 'cognitive dysfunction':ti,ab,kw OR 'vascular dementia':ti,ab,kw OR 'mini mental state':ti,ab,kw OR moca:ti,ab,kw OR 'cognition disorder':ti,ab,kw  AND  'cerebrovascular accident'/exp OR 'ischemic stroke'/exp OR 'lacunar stroke'/exp OR 'cardioembolic stroke'/exp OR 'brainstem stroke'/exp OR 'brain infarction'/exp OR 'transient ischemic attack'/exp OR 'cerebrovascular disease'/exp OR 'brain ischemia'/exp OR 'cerebral artery disease'/exp OR 'brain embolism'/exp OR 'brain hemorrhage'/exp OR 'stroke':ti,ab,kw OR 'brain lesion':ti,ab,kw OR 'cerebral vascular disorder':ti,ab,kw OR 'brain infarct':ti,ab,kw OR 'hemorrhagic stroke':ti,ab,kw OR 'embolic stroke':ti,ab,kw OR 'thrombotic stroke':ti,ab,kw OR 'ischemic stroke':ti,ab,kw OR 'stroke rehabilitation':ti,ab,kw |
| Scopus | | |
|  | Filters applied | Human subjects, adult subjects, language (English), multicenter study, observational study |
|  | Search | ( ( TITLE-ABS-KEY ( stroke ) OR TITLE-ABS-KEY ( apoplexy ) OR TITLE-ABS-KEY ( "brain infarction" ) OR TITLE-ABS-KEY ( "cerebral infarction" ) ) ) AND ( ( TITLE-ABS-KEY ( "blood pressure" ) OR TITLE-ABS-KEY ( hypertension ) OR TITLE-ABS-KEY ( hypotension ) ) ) AND ( ( TITLE-ABS-KEY ( "cognitive impairment" ) OR TITLE-ABS-KEY ( "cognitive decline" ) OR TITLE-ABS-KEY ( "cognitive dysfunction" ) OR TITLE-ABS-KEY ( "cognitive defect" ) OR TITLE-ABS-KEY ( dementia ) ) ) AND ( ( TITLE-ABS-KEY ( observational ) OR TITLE-ABS-KEY ( "cross-sectional" ) OR TITLE-ABS-KEY ( "cohort" ) OR TITLE-ABS-KEY ( "prospective" ) OR TITLE-ABS-KEY ( "retrospective" ) ) ) |
